# Comparative Genomic Analysis of Members of the Genera *Methanosphaera* and *Methanobrevibacter* Reveals Distinct Clades with Specific Potential Metabolic Functions

**DOI:** 10.1155/2018/7609847

**Published:** 2018-08-19

**Authors:** Anja Poehlein, Dominik Schneider, Melissa Soh, Rolf Daniel, Henning Seedorf

**Affiliations:** ^1^Genomic and Applied Microbiology and Göttingen Genomics Laboratory, Georg-August University, Göttingen, Germany; ^2^1 Research Link, Temasek Life Sciences Laboratory, Singapore 117604; ^3^National University of Singapore, Department of Biological Sciences, Singapore 117604

## Abstract

*Methanobrevibacter* and *Methanosphaera* species represent some of the most prevalent methanogenic archaea in the gastrointestinal tract of animals and humans and play an important role in this environment. The aim of this study was to identify genomic features that are shared or specific for members of each genus with a special emphasis of the analysis on the assimilation of nitrogen and acetate and the utilization of methanol and ethanol for methanogenesis. Here, draft genome sequences of *Methanobrevibacter thaueri* strain DSM 11995^T^, *Methanobrevibacter woesei* strain DSM 11979^T^, and *Methanosphaera cuniculi* strain 4103^T^ are reported and compared to those of 16 other *Methanobrevibacter* and *Methanosphaera* genomes, including genomes of the 13 currently available types of strains of the two genera. The comparative genome analyses indicate that among other genes, the absence of molybdopterin cofactor biosynthesis is conserved in *Methanosphaera* species but reveals also that the three species share a core set of more than 300 genes that distinguishes the genus *Methanosphaera* from the genus *Methanobrevibacter*. Multilocus sequence analysis shows that the genus *Methanobrevibacter* can be subdivided into clades, potentially new genera, which may display characteristic specific metabolic features. These features include not only the potential ability of nitrogen fixation and acetate assimilation in a clade comprised of *Methanobrevibacter* species from the termite gut and *Methanobrevibacter arboriphilus* strains but also the potential capability to utilize ethanol and methanol in a clade comprising *Methanobrevibacter wolinii* strain DSM 11976^T^, *Mbb.* sp. AbM4, and *Mbb. boviskoreani* strain DSM 25824^T^.

## 1. Introduction

The microbial ecology of the intestinal tract of humans and animals has been subject to extensive research in recent years, and it is becoming increasingly evident that commensal intestinal microbes have a strong impact on host physiology and well-being. One microbial group, the methanogenic archaea (methanogens), has been of particular interest in this regard. Methanogens have not only been implicated in greenhouse gas emission from livestock animals [1, 2] but several studies have also linked these microorganisms to specific bodyweight phenotypes in humans [3] and to differences in feed conversion efficiency in ruminants [4, 5].

The majority of the rumen and intestinal methanogens appear to belong to two of the seven known orders, the Methanobacteriales [6] and the recently described Methanomassiliicoccales [7]. Two genera of the order Methanobacteriales, *Methanobrevibacter* and *Methanosphaera*, comprise a large proportion of intestinal and rumen methanogenic archaea [8, 9]. Species of the genus *Methanobrevibacter* have been isolated from not only a wide range of different environments, including invertebrate and vertebrate guts, but also non-host-associated environments. The majority of the members of the genus *Methanobrevibacter* grow primarily hydrogenotrophically [10], using CO_2_ and H_2_ as substrates. A few strains and/or species have been shown by cultivation-independent methods to be the predominant in specific intestinal environments, such as *Mbb. smithii* from the human gut [8] or *Mbb. ruminantium*, and *Mbb. gottschalkii* from the rumen of sheep and cows [11]. The former three species have gained considerable attention in the analysis of the human and rumen microbiome, but there are also *Mbb.* isolates that are less well investigated by cultivation-independent studies, for example, *Mbb. thaueri* strain DSM 11995^T^ and *Mbb. woesei* strain DSM 11976^T^ *. Mbb. thaueri* strain DSM 11995^T^ was isolated from cow feces while *Mbb. woesei* strain DSM 11976^T^ had been isolated from goose feces [12]. The 16S rRNA gene of either of the two species shares the highest sequence identity to *Mbb. smithii* strain DSM 861^T^, *Mbb. gottschalkii* strain DSM 11977^T^, and *Mbb. millerae* strain DSM 16643^T^ [12–15]. Both species have been detected in a few cultivation-independent studies on fecal and rumen samples (for *Mbb. thaueri*, see [16, 17] and for *Mbb. woesei*, see [18–20]), but besides their original description, there is very little information on the physiology and ecology of these two species.

The genus *Methanosphaera* is less well characterized than the genus *Methanobrevibacter*, and only few species have been isolated or detected by cultivation-independent methods. Two species, *Msp. stadtmanae* DSM 3091^T^ and *Msp. cuniculi* DSM 4103^T^, are currently the only formally described *Methanosphaera*-type species [21, 22]. The first genome-sequenced *Methanosphaera* species, *Msp. stadtmanae* DSM3091^T^, was isolated from human feces and has been shown to be restricted to growth on methanol and hydrogen under *in vitro* conditions [22]. This substrate restriction can to some extent be considered to be a typical trait of the genus *Methanosphaera*, but its genetic basis remained poorly understood until the genome of *Msp. stadtmanae* strain DSM 3091^T^ was analyzed [23]. Comparative genome analysis revealed that most genes for biosynthesis of the molybdopterin cofactor (Moco), the cofactor of formylmethanofuran dehydrogenase (fmd), are absent from the genome of this methanogen. This explained why *Msp. stadtmanae* strain DSM3091^T^ is not able to grow by disproportionation of methanol nor of CO_2_ and hydrogen as substrates. Moreover did the analysis reveal that the genome encodes four homologues of each subunit of the methanol : coenzyme-M methyltransferase (mtaABC) [23]. The MtaABC proteins form the key enzyme complex for the utilization of methanol and had before primarily been detected in species of the order Methanosarcinales.

The second genome of a genus *Methanosphaera* representative, *Methanosphaera* sp. WGK6, has only recently become available. This methanogen's genome sequence is to large extent nearly identical to that of *Msp. stadtmanae* DSM 3091^T^, but some of the few genomic differences result in phenotypic differences between the two species. This regards mainly two genes, putative alcohol (walC) and aldehyde (walD) dehydrogenases [24]. These two genes were apparently acquired via horizontal gene transfer and seem to enable *Msp.* sp. WGK6 to utilize ethanol as substrate for methanogenesis (in addition to its ability to grow on methanol and hydrogen) [24]. Homologues of walC and walD genes have also been detected in genomes of other methanogens but have not been investigated systematically.

Here, draft genomes of *Mbb. thaueri* strain DSM 11995^T^, *Mbb. woesei* strain DSM 11979^T^, and *Msp. cuniculi* strain DSM 4103^T^ (a draft genome of *M. cuniculi* DSM 4103^T^ of similar quality was also recently published by Gilmore et al. [25]) are presented and compared to those of all other currently available *Methanosphaera*- and *Methanobrevibacter*-type strain genomes. The comparative analyses aim at investigating and identifying some of the distinctive features of the *Methanobrevibacter* and *Methanosphaera* genomes that allow differentiating the two closely related genera. The major emphasis was set on key genes that may have large impact on the overall physiology of the analyzed species, such as those involved in substrate utilization and nitrogen and acetate assimilation.

## 2. Materials and Methods

### 2.1. Cultivation of Microorganism and DNA Extraction

*Mbb. thaueri* DSM 11995^T^, *Mbb. woesei* DSM 11979^T^, and *Msp. cuniculi* DSM 4103^T^ were obtained from the Deutsche Sammlung von Mikroorganismen und Zellkulturen (DSMZ), Braunschweig, Germany. Genomic DNA was ordered by the DSMZ (Braunschweig) or was isolated using the MasterPure complete DNA purification kit (Epicentre, Madison, WI, USA).

### 2.2. Genome Sequencing

Extracted DNA was used to generate Illumina-shotgun libraries (Nextera_XT) according to the manufacturer's protocol (Illumina, San Diego, CA, USA). Sequencing was conducted using a MiSeq and Miseq Reagent kit v3 (2 × 300 bp paired end) as recommended by the manufacturer (Illumina). Trimmomatic 0.32 [26] was used to filter low-quality reads and for clipping of adapter contaminations. The assembly was performed with the SPAdes genome assembler software 3.11 [27]. Coverages were determined using QualiMap version 2.1 [27–29], and automatic annotation was performed using the software tool PROKKA [30], and data analysis was partly performed using the IMG/ER system (Integrated Microbial Genomes & Microbiomes) [31]. The quality and the completeness of the draft genomes have been validated with CheckM [32].

### 2.3. Multilocus Sequence Analysis

For multilocus sequence analysis (MLSA), total protein sequences from 19 genomes were extracted from the corresponding GenBank files using cds_extractor.pl v0.6 [33] and used for downstream analysis with an in-house pipeline at the Goettingen Genomics Laboratory [34]. In detail, proteinortho version 5 (default specification: blast = blastp v2.2.24, *E* value = 1*e* − 10, alg.-conn. = 0.1, coverage = 0.5, percent_identity = 50, adaptive_similarity = 0.95, inc_pairs = 1, inc_singles = 1, selfblast = 1, and unambiguous = 0) [35] was used to generate clusters of orthologue groups, inparalogues were removed, and MUSCLE [36] used to align the remaining sequences and poorly aligned positions were automatically filtered from the alignments using Gblocks [37]. A maximum-likelihood tree from 574 orthologues was inferred with 500 bootstraps with RAxML [38]. A phylogenetic tree was inferred with neighbour joining and 500 bootstraps. PO_2_MLSA.py is available at GitHub (https://github.com/jvollme). Visualization was done using Proteinortho results and DNAPlotter [39]. The Venn diagram of the different clades was plotted using po2group_stats (v0.1.1) [33].

#### 2.3.1. Analysis of 16S rRNA Genes

Aligned 16S rRNA gene sequences were selected from the ARB-compatible rumen and intestinal methanogen database (RIM-DB) [14, 40]. Aligned sequences were exported in PHYLIP format to construct phylogenetic trees using all available base positions. Maximum-likelihood phylogenetic trees based on aligned archaeal 16S rRNA gene sequences were generated using RAxML version 7.3.0 [38]. Unless stated otherwise, the parameters “-m GTRGAMMA -# 500 -f a -× 2 -p 2” were used.

### 2.4. Nucleotide Sequence Accession Number

The annotated genomes of *Mbb. thaueri* DSM 11995^T^ and *Mbb. woesei* DSM 11979^T^ have been deposited at DDBJ/EMBL/GenBank under the accession MZGS00000000 and MZGU00000000, respectively. The versions described in this paper are versions MZGS01000000 and MZGU01000000, respectively. The annotated genome of *Msp. cuniculi* DSM 4103^T^ has been deposited at DDBJ/EMBL/GenBank under the accession LWMS00000000. The version described in this paper is version LWMS01000000.

## 3. Results

### 3.1. General Genome Features

The *Methanosphaera cuniculi* strain DSM 4103^T^ draft genome has been assembled into 48 contigs, with a N50 of 111,976 bp. The GC content of the draft genome is 28%, which is similar to the G + C content of the other two published *Methanosphaera* genome sequences. The genomes of *Mbb. thaueri* strain DSM 11995^T^ and *Mbb. woesei* strain DSM 11979^T^ were assembled into 39 contigs and 10 contigs with N50 of 178.217 bp and 240.239 bp, respectively. Plasmids were not detected from the assembled contigs in either of the two draft genomes. General features of the of *Mbb. thaueri* DSM 11995^T^, *Mbb. woesei* strain DSM 11979^T^, and *Methanosphaera cuniculi* strain DSM 4103^T^ genomes and comparison with other *Methanobrevibacter* and *Methanosphaera* strains are shown in Table 1, and results of CheckM analysis (including all genomes used in the analysis) are shown in Table S1. Circular representation of the three genomes is shown in supporting Figures S1 and S2.

### 3.2. Comparative Analysis of *Methanobrevibacter* Genomes

**Figure 1:**
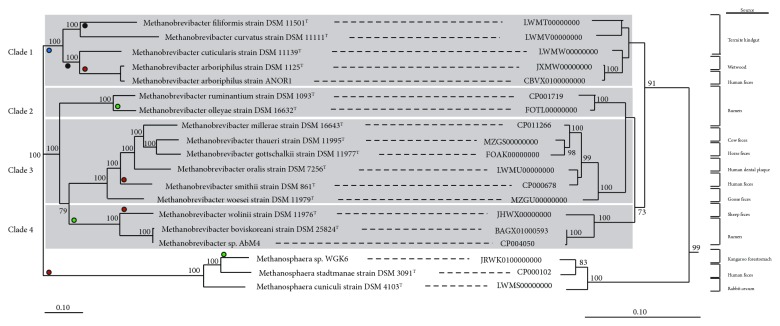
Single- and multilocus sequence analysis of *Methanobrevibacter* and *Methanosphaera* species. A maximum likelihood tree (left) of 16 *Methanobrevibacter* and three *Methanosphaera* genomes was inferred with 500 bootstraps with RAxML and visualized with Dendroscope. Phylogeny of *Methanobrevibacter* and *Methanosphaera* based on the 16S rRNA gene is shown on the right. The tree was resampled 500 times, and only bootstrap values ≥ 70% are shown. The 16S rRNA tree was rooted with five *Methanobacterium* sequences. The scale bar indicates 0.10 inferred nucleotide substitutions per position. Red-colored dots indicate the presence of mtaABC genes in the species/clade, green-colored dots indicate the presence of walB and walC gene homologues (potential utilization of ethanol), blue-colored dots indicate the presence of nitrogenase genes, and black-colored dots indicate the presence of carbon monoxide/acetyl CO-DH genes (see also Table S3 for details).

Sixteen different *Methanobrevibacter* and three *Methanosphaera* genomes were included for the comparative analyses. The *Methanobrevibacter* strains had been isolated from a range of different intestinal environments, for example, bovine and ovine rumen [12, 41], human gut [42], and termite hindguts [43, 44], but include also the genome of the non-host-associated *Mbb. arboriphilus* strain DSM 1125^T^ [45, 46]. Results of the single- and multilocus analyses are shown in Figure 1. Both analyses revealed clear separation between the two genera.

**Figure 2:**
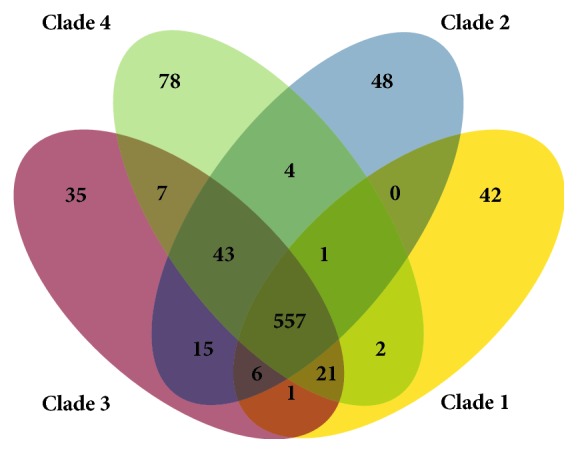
Pan/core genome analysis of four different *Methanobrevibacter* clades. Venn diagram showing the numbers of orthologous genes (OGs) in the core, dispensable, and specific genome of compared strains. Ortholog detection was done with the Proteinortho software (blastp) with a similarity cut-off of 50% and an *E* value of 1*e* − 10. The total numbers of genes and paralogs are depicted under the corresponding species name. Open-reading frames that were classified as pseudogenes were not included in this analysis. See also Table S3 for details on shared genes.

The MLSA also supports distinct clades within the genus *Methanobrevibacter* (Figure 1). There is limited association of the observed clades with a specific host origin, for example, some species from the rumen form distinct clades while others cluster with *Methanobrevibacter* species isolated from other hosts. A BLAST-based analysis of each clade identifies genes specific for clades and genes that are shared between them (Figure 2). Among the clade-specific genes of clade 1 are catalase, nitrogenase, and also a tRNA-specific endonuclease (VapC), but most other clade-specific genes of clade 1 and the other clades are without functional annotation and await further characterization.

It was also investigated whether certain metabolic traits could be associated with specific clades as pointed out in Figure 1. This concerns mainly the metabolism of alcohols and acetate assimilation. Carbon monoxide/acetyl-CoA-synthetase complex (CODH-ACS) is—in addition to other enzymes—important for acetate assimilation, and its presence is one prerequisite for autotrophic growth. CODH-ACS is found in non-host-associated and autotrophic methanogens, for example, *Methanothermobacter* species [47–49], and is detected in four out of five clade 1 species, but gene homologues of the enzyme appear to be absent from the genomes of methanogens in clades 2–4.

The potential utilization of alcohols, specifically methanol and ethanol, is also less clearly distributed among the 19 genomes (and the four different clades) and there are currently no known *Methanobrevibacter* species that are restricted to growth on H_2_ and methanol/ethanol [10]. Genes for methanol utilization of the *Methanosphaera/Methanosarcina* type (mtaABC) are distributed broadly within the genus *Methanobrevibacter* as outlined in Figure 1 and have also already been reported for some strains [46, 50]. Physiological characterizations of the strains that harbor mtaABC genes (*Mbb. arboriphilus* strains DSM 1125^T^ and ANOR1, *Mbb. smithii* strain DSM 861^T^, *Mbb. wolinii* strain DSM 11976^T^) is still outstanding to confirm that these species are capable of growth on methanol/methanol-hydrogen.

The utilization of ethanol by *Methanobrevibacter* species has only recently gained additional attention and it has been shown that *Mbb.* sp. AbM4 is capable of growth in the absence of hydrogen but in presence of methanol/ethanol [51, 52]. Details regarding the exact metabolism remain speculative, but the walC and walD genes recently identified in *Msp.* WGK6 [24] are also present in *Mbb.* sp. AbM4 and in the closely related *Mbb. boviskoreani* strain DSM 25824^T^, *Mbb. olleyae* strain DSM 16632^T^, and *Mbb. wolinii* strain DSM11976^T^ conferring this metabolic trait potentially to the entire clade. *Mbb. olleyae* strain DSM 16632^T^ is currently the only *Mbb*. species outside this clade that is harboring the walCD genes.

Only few additional genome sequences of *Methanobrevibacter* isolates other than for *Mbb. smithii* are currently available, but the clade-distinguishing features appear to be conserved in these isolates as well (see Table S2 for general genome features and Table S3 for shared genes).

### 3.3. Shared and Distinctive Genome Features in the Genomes of *Methanosphaera* Species

**Figure 3:**
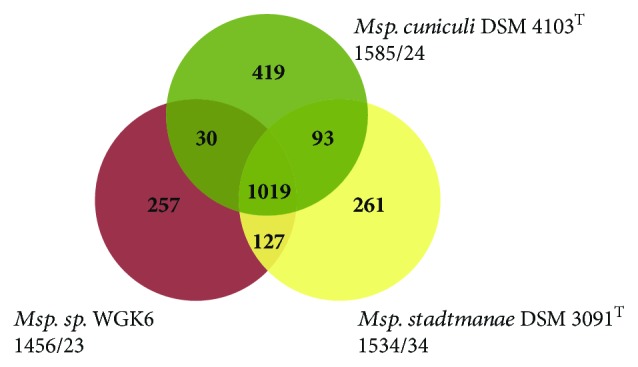
Pan/core genome analysis of three different *Methanosphaera* species. Venn diagram showing the numbers of orthologous genes (OGs) in the core, dispensable, and specific genome of compared strains. Ortholog detection was done with the Proteinortho software (blastp) with a similarity cut-off of 50% and an *E* value of 1*e* − 10. The total numbers of genes and paralogs are depicted under the corresponding species name. Open-reading frames that were classified as pseudo genes were not included in this analysis. See also Table S3 for details on shared genes.

The currently known *Methanosphaera* species are characterized by their limited ability to use substrates for methanogenesis, for example, *Msp. stadtmanae* and *Msp. cuniculi* are able to utilize only methanol and hydrogen for growth, *Msp.* sp. WKG6 can also utilize ethanol, but none of the species is capable of utilizing hydrogen and CO_2_ or formate like organisms from closely related genera. The analysis of the *Msp. stadtmanae* genome helped explaining some of the distinctive physiological features of this methanogen, but comparative genome analysis with other closely related *Methanosphaera* and *Methanobrevibacter* species was not possible at that time due to the lack of sequenced genomes. After more than a decade, several *Methanosphaera* and *Methanobrevibacter* genomes have become available [24, 41, 46, 50, 53–58] and comparative analyses allow determining whether certain traits are species specific or shared by members of either or both genera. Comparative genome analysis reveals that some of the observations made for the *Msp. stadtmanae* genome appear to be consistent for all three available *Methanosphaera* genomes. Overall, more than 1000 genes are shared among all three *Methanosphaera* species (Figure 3), but the comparison with *Methanobrevibacter* genomes shows that several key genes are missing. Notably, this concerns the lack of genes for molybdopterin cofactor biosynthesis, energy-converting hydrogenase A (ehaA-Q), formate dehydrogenase (fdh), and methyl-coenzyme M reductase isoenzyme 1 mcrABCG (only genes encoding isoenzyme II are present (mrtABDG)). Despite the presence of formylmethanofuran dehydrogenase genes, it is not possible for *Methanosphaera* species to produce a functional enzyme due to the absence of the molybdopterin cofactor. The absence of functional formylmethanofuran dehydrogenase leads to the inability of these methanogens to grow hydrogenotrophically or to disproportionate methanol. The absence of Moco biosynthesis also seems to be specific to *Methanosphaera* species as all of the analyzed *Methanobrevibacter* genomes are capable of synthesizing this cofactor.

One of the noteworthy differences between the known *Methanosphaera* species is the capability of *Msp.* WGK6 to utilize ethanol as an electron donor for methanogenesis. The recently reported alcohol dehydrogenase and aldehyde dehydrogenase (NL43_02835, NL43_02830) in the genome of *Methanosphaera* sp. WGK6 were not detected in the *Msp. stadtmanae* genome (as previously reported by Hoedt et al., 2016) or the *Msp. cuniculi* genome [25], indicating that this may be a trait of *Msp*. WGK6 that is shared with other less-related methanogens.

## 4. Discussion

The presented comparative genome analysis reveals insights into the genome content of different *Methanobrevibacter* species and how it compares to that of closely related *Methanosphaera* species. The analysis suggests that phenotypic variation among *Methanobrevibacter* strains may be larger than previously assumed. The comparative analysis indicates the presence of genes for methanol (in four *Mbb*. genomes) and ethanol utilization (in four *Mbb*. genomes) in the sixteen analyzed *Methanobrevibacter* genomes, with the *Mbb. wolinii* genome harboring both walCD and mtaABC gene homologues. More physiological analysis will be required to determine if the detected genes are conferring the ability to utilize ethanol/methanol to all these strains. The ability of *Methanobrevibacter* strains to utilize alcohols may not have been investigated in an in-depth manner previously as this trait is more typically associated with other orders of methanogens, but some studies suggest that the genes may in some cases also not be functional or may have unknown different functions, for example, growth of *Mbb. smithii* on methanol was not detected despite the presence of mtaABC genes (growth on methanol but not methanol and hydrogen was tested) [59]. It is noteworthy that all three sequenced strains of the *Mbb. wolinii* clade harbor the genes for ethanol utilization, which could point to a specific ecological role of the species in this clade; however, there are only few studies that have detected significant numbers of either of these three species in a natural environment [5, 60] making it currently difficult to determine potential cooccurrences or specific syntrophic interactions with other microorganisms.

The presence of genes that could contribute to autotrophic growth, for example, nitrogenase and CODH-ACS in clade 1 containing *Mbb.* species from termites and in *Mbb. arboriphilus* strain, has been reported previously [46], and these genes appear to be absent from species clades 2 to 4. It can only be speculated why *Mbb.* species of clade 1 did not undergo the same loss of these key genes like their counterparts in the clades that have primarily been isolated from the vertebrate intestinal tract. However, auxotrophy of some *Mbb.* species may be the result of a close symbiotic interaction with other microorganisms (and potentially the host) that provide favorable growth conditions as well as ammonium and acetate for the methanogen. It is also noteworthy that *Mbb. curvatus* in the “autotrophic clade” has nitrogenase genes, but apparently, no CODH complex genes and may therefore require externally supplied acetate. This could represent an intermediate stage between the potentially autotrophic strains and the other clades and/or may be an adaptation to a specific niche.

In addition to differences between species, there is currently only little information on strain diversity within a species but studies indicate that there may be considerable differences as shown for *Mbb. smithii* and *Mbb. arboriphilus* [46, 61]. However, undertaking pan-genome approaches, such as the one for *Mbb. smithii* by Hansen et al. [61], requires substantial cultivation efforts as there are only few strains available for each of the described methanogen species. As development of sequencing technologies continues to advance, it may also become feasible to obtain high-quality closed genomes from low amounts of starting DNA or through metagenomic approaches. Having such genomes will allow greater certainty in determining the presence and absence of specific genome features and will also enable detection of small genomic differences between strains that may go unnoticed in draft genomes.

Lastly, the results of our analyses corroborate the hypothesis that the absence of molydopterin cofactor biosynthesis is a characteristic trait shared by members of the genus *Methanosphaera*, while all sequenced *Methanobrevibacter* genomes seem to encode genes for Moco biosynthesis. However, it also needs to be considered that *Methanosphaera* species encode the genes for formylmethanofuran dehydrogenase and other genes required for hydrogenotrophic or methylotrophic methanogenesis [62, 63]. The presence of these genes may indicate that *Methanosphaera* species may still be able to utilize the aforementioned methanogenic pathways, if molybdopterin cofactor is present and taken up by the methanogen (as suggested by Fricke et al. 2006). Metatranscriptional profiling could be used to determine, if fmd and other genes for hydrogenotrophic growth are expressed at all in *Methanosphaera* or specifically in the absence and presence of Moco or Moco-synthesizing microorganisms. It needs to be emphasized that the analyses of this study are based on the currently available three genomes of the three *Methanosphaera* species that have been isolated from three different host species [23–25]. It provides strong evidence that the physiology will be similar for other *Msp.* species in other environments, but it can also not be ruled out that other *Msp.* species exist that harbor genes for Moco biosynthesis and that grow hydrogenotrophically or methylotrophically. The ability of *Msp*. sp. WGK6 to utilize ethanol does indicate that more phenotypic variation exists among *Methanosphaera* species and that further isolation and characterization of new *Msp*. species is necessary.

## 5. Conclusion

The presented study includes genomes of all currently available types of strain (and other selected isolate) of *Methanobrevibacter* and *Methanosphaera* genomes and allows comprehensive insights into the genera *Methanobrevibacter* and *Methanosphaera*. The analyses reveal that distinct clades within the genus *Methanobrevibacter* exist and that the lack of molybdopterin cofactor biosynthesis may be a specific trait for the genus *Methanosphaera*. Additional isolates and further physiological and genomic analyses will be required to determine if division of the genus *Methanobrevibacter* in more than one genus could be justified. The primary use of the type of strains (and other isolates) for the analysis in this study does warrant access of the scientific community to most of the analyzed *Methanobrevibacter* and *Methanosphaera* isolates and will facilitate testing of the predicted physiological phenotypes.

## Figures and Tables

**Table 1:** General features of Methanobrevibacter and Methanosphaera genomes.

	Size (bp)	GC content (%)	Coding percentage (%)	CDS (pseudo)	Genes (pseudo)	rRNA	tRNA	Accession	Contigs/scaffolds	CRISPR region	Original strain ID
*Methanobrevibacter filiformis* DSM 11501^T^	2,606,143	26.99	69.94	1933	1965	3	29	LWMT00000000	237	6	RFM-3
*Methanobrevibacter curvatus* DSM 11111^T^	2,414,608	25.72	70.65	1969	2004	4	31	LWMV00000000	187	5	RFM-2
*Methanobrevibacter cuticularis* DSM 11139^T^	2,608,702	26.79	68.12	2061	2094	3	30	LWMW00000000	120	6	RFM-1
*Methanobrevibacter arboriphilus* DSM 1125^T^	2,445,031	25.44	74.96	1963	2005	5	37	JXMW00000000	40	9	DH1
*Methanobrevibacter arboriphilus* ANOR1	2,221,072	25.53	74.02	1993	2038	7	35	CBVX000000000	5	3	ANOR1
*Methanobrevibacter ruminantium* DSM 1093^T^	2,937,203	32.64	78.12	2217	2283 (5)	8	53	CP001719	1	4	M1
*Methanobrevibacter olleyae* DSM 16632^T^	2,122,444	26.87	76.85	1813	1854	4	33	FOTL00000000	49	4	KM1H5-1P
*Methanobrevibacter millerae* DSM 16643^T^	2,725,667	36.54	89.32	2383	2467	4	77	CP011266	1	1	ZA-10
*Methanobrevibacter thaueri* DSM 11995^T^	2,243,115	36.87	87.52	2138	2171	2	31	MZGS00000000	39	4	CW
*Methanobrevibacter gottschalkii* DSM 11977^T^	1,879,371	30.02	88.01	1845	1889	7	34	FOAK00000000	19	NA	HO
*Methanobrevibacter oralis* DSM 7256^T^	2,110,861	27.73	84.49	2036	1994	9	32	LWMU00000000	99	2	ZR
*Methanobrevibacter smithii* DSM 861^T^	1,853,160	31.03	90.31	1795	1841	8	36	CP000678	1	1	PS
*Methanobrevibacter woesei* DSM 11979^T^	1,543,150	29.90	89.61	1581	1614	2	31	MZGU00000000	10	2	GS
*Methanobrevibacter wolinii* DSM 11976^T^	2,041,814	24.21	75.94	1700	1747	8	36	JHWX00000000	32	2	SH
*Methanobrevibacter boviskoreani* DSM 25824^T^	2,045,801	28.98	78.00	1756	1799	4	36	BAGX00000000	54	2	JH1
*Methanobrevibacter* sp. AbM4	1,998,189	29.04	76.7	1671	1716	49	36	CP004050	1	6	N.A.
*Methanosphaera* sp. WGK6	1,729,155	27.70	78.05	1456	1616 (114)	3	42	JRWK00000000	37	1	N.A.
*Methanosphaera stadtmanae* DSM 3091^T^	1,767,403	27.63	84.10	1534	1592 (2)	12	42	CP000102	1	4	MCB-3
*Methanosphaera cuniculi* DSM 4103^T^	1,881,497	28.08	81.36	1585	1629	5	39	LWMS00000000	48	0	1R-7

## Data Availability

The data used to the support the findings of this study are availabe in the supplemental information or -as in the case of the genomes- are downloadable using the accession numbers provided in Section 2.4.

## Supplementary Materials

Supplementary 1 Figure S1: circular display of the *Mbb. thaueri* strain DSM 11995^T^ *and Mbb. woesei* strain DSM 11979^T^ genomes and comparison with other *Methanobrevibacter* genomes. The *Mbb. thaueri* strain DSM 11995^T^ and the *Mbb. woesei* strain DSM 11979^T^ genomes are shown in panels A and B, respectively. The genes encoded by the leading and the lagging strand of the respective species are shown in circles 1 and 2, and rRNA (pink) and tRNA (green) encoding genes are shown in circle 3. The presence of orthologous genes (red: high similarity; orange: medium similarity; yellow: low similarity; see color code below) is indicated for the other *Methanobrevibacter* genomes (circles 4 to 19) in comparison to that of the respective *Methanobrevibacter* genome. The two innermost plots represent the GC content and the GC skew (circle 20 and 21). Color code according to *E* values of the blastp analysis performed using Proteinortho4.26. Gray: 1*e* − 20 to 1; light yellow: 1*e* − 21 to 1*e* − 50; gold: 1*e* − 51 to 1*e* − 90; light orange: 1*e* − 91 to 1*e* − 100; orange: 1*e* − 101 to 1*e* − 120; red: >1*e* − 120.

Supplementary 2 Figure S2: circular display of the *Msp. cuniculi* strain DSM 4103^T^ genome and comparison with the genomes of *Msp. stadtmanae* strain DSM 3091^T^ and *Msp.* sp. WGK6. The genes encoded by the leading and the lagging strand of *Msp. cuniculi* strain DSM 4103^T^ are shown in circles 1 and 2, and rRNA (pink) and tRNA (green) encoding genes are shown in circle 3. The presence of orthologous genes (red: high similarity; orange: medium similarity; yellow: low similarity; see color code below) is indicated for the genomes of *Msp. stadtmanae* strain DSM 3091^T^ and *Msp*. sp. WGK6 (circles 4 and 5 in comparison to the *Msp. cuniculi* strain DSM 4103^T^ genome). The two innermost plots represent the GC content and the GC skew (circles 6 and 7). Color code according to *E* values of the blastp analysis performed using Proteinortho4.26. Gray: 1*e* − 20 to 1; light yellow: 1*e* − 21 to 1*e* − 50; gold: 1*e* − 51 to 1*e* − 90; light orange: 1*e* − 91 to 1*e* − 100; orange: 1*e* − 101 to 1*e* − 120; red: >1*e* − 120.

Supplementary 3 Table S1: CheckM analysis to determine the completeness of genomes. Table S1A shows results from analysis of genomes from the main manuscript while Table S1B lists the results for genomes mainly mentioned in the supporting information. Table S2: general features of additional *Methanobrevibacter* genomes. General features of genomes mainly mentioned in the supporting information. Table S3: table of shared and unshared *Methanobrevibacter* and *Methanosphaera* genes. Highlighted in colors corresponding to those of Figure 1 are genes for CODH-ACS complex, nitrogenase, methanol, and ethanol utilization. Annotations were taken from the *Mbb. arboriphilus* DSM1125^T^ genome.
